# Integrated spin-wave quantum memory

**DOI:** 10.1093/nsr/nwae161

**Published:** 2024-05-01

**Authors:** Tian-Xiang Zhu, Ming-Xu Su, Chao Liu, Yu-Ping Liu, Chao-Fan Wang, Pei-Xi Liu, Yong-Jian Han, Zong-Quan Zhou, Chuan-Feng Li, Guang-Can Guo

**Affiliations:** CAS Key Laboratory of Quantum Information, University of Science and Technology of China, Hefei 230026, China; CAS Center for Excellence in Quantum Information and Quantum Physics, University of Science and Technology of China, Hefei 230026, China; CAS Key Laboratory of Quantum Information, University of Science and Technology of China, Hefei 230026, China; CAS Center for Excellence in Quantum Information and Quantum Physics, University of Science and Technology of China, Hefei 230026, China; CAS Key Laboratory of Quantum Information, University of Science and Technology of China, Hefei 230026, China; CAS Center for Excellence in Quantum Information and Quantum Physics, University of Science and Technology of China, Hefei 230026, China; CAS Key Laboratory of Quantum Information, University of Science and Technology of China, Hefei 230026, China; CAS Center for Excellence in Quantum Information and Quantum Physics, University of Science and Technology of China, Hefei 230026, China; Hefei National Laboratory, University of Science and Technology of China, Hefei 230088, China; CAS Key Laboratory of Quantum Information, University of Science and Technology of China, Hefei 230026, China; CAS Center for Excellence in Quantum Information and Quantum Physics, University of Science and Technology of China, Hefei 230026, China; CAS Key Laboratory of Quantum Information, University of Science and Technology of China, Hefei 230026, China; CAS Center for Excellence in Quantum Information and Quantum Physics, University of Science and Technology of China, Hefei 230026, China; CAS Key Laboratory of Quantum Information, University of Science and Technology of China, Hefei 230026, China; CAS Center for Excellence in Quantum Information and Quantum Physics, University of Science and Technology of China, Hefei 230026, China; Hefei National Laboratory, University of Science and Technology of China, Hefei 230088, China; CAS Key Laboratory of Quantum Information, University of Science and Technology of China, Hefei 230026, China; CAS Center for Excellence in Quantum Information and Quantum Physics, University of Science and Technology of China, Hefei 230026, China; Hefei National Laboratory, University of Science and Technology of China, Hefei 230088, China; CAS Key Laboratory of Quantum Information, University of Science and Technology of China, Hefei 230026, China; CAS Center for Excellence in Quantum Information and Quantum Physics, University of Science and Technology of China, Hefei 230026, China; Hefei National Laboratory, University of Science and Technology of China, Hefei 230088, China; CAS Key Laboratory of Quantum Information, University of Science and Technology of China, Hefei 230026, China; CAS Center for Excellence in Quantum Information and Quantum Physics, University of Science and Technology of China, Hefei 230026, China; Hefei National Laboratory, University of Science and Technology of China, Hefei 230088, China

**Keywords:** quantum memory, integrated optics, quantum network, quantum optics

## Abstract

Photonic integrated quantum memories are essential for the construction of scalable quantum networks. Spin-wave quantum storage, which can support on-demand retrieval with a long lifetime, is indispensable for practical applications, but has never been demonstrated in an integrated solid-state device. Here, we demonstrate spin-wave quantum storage based on a laser-written waveguide fabricated in a ^151^Eu^3+^:Y_2_SiO_5_ crystal, using both the atomic frequency comb and noiseless photon-echo protocols. Qubits encoded with single-photon-level inputs are stored and retrieved with a fidelity of ${94.9\%\pm 1.2\%}$, which is far beyond the maximal fidelity that can be obtained with any classical device. Our results underline the potential of laser-written integrated devices for practical applications in large-scale quantum networks, such as the construction of multiplexed quantum repeaters in an integrated configuration and high-density transportable quantum memories.

## Teaser

Single-photon-level light is stored as a spin-wave excitation in a laser-written waveguide, unlocking its practical applications in quantum repeaters and transportable quantum memories.

## INTRODUCTION

Photonic quantum memories, which can faithfully store photonic qubits, are the core devices to overcome channel losses in long-distance quantum networks [1,2]. Photonic quantum memories have been demonstrated with single atoms [3,4], atomic gases [5–8] and rare-earth-ion-doped crystals (REICs) [9–14], and integrated operations have been demonstrated in REICs based on various fabrication techniques [15–21]. However, all previous demonstrations of integrated quantum memories for light are limited to storage in optically excited states [15–20,22,23], which does not support on-demand retrieval with continuously adjustable storage times, and the storage time is fundamentally limited by the excited-state lifetime. Spin-wave storage, which utilizes spin-wave excitation for photonic storage [3–8,24–28], could enable on-demand retrieval with a storage time extended to the spin coherence lifetime. It has been demonstrated in integrated solid-state devices for classical light [29–32], rather than single-photon-level inputs, due to the formidable challenges of suppressing the noise inside the integrated structures and preserving coherent properties of rare-earth ions during the fabrication process [21].

Here, we demonstrate an integrated spin-wave quantum memory using a laser-written waveguide fabricated in a ^151^Eu^3+^:Y_2_SiO_5_ crystal. A ^151^Eu^3+^:Y_2_SiO_5_ crystal is a unique material for quantum information processing that has the longest spin coherence lifetime (6 h) [33] and the longest storage lifetime for light (1 h) reported so far [2]. We employ direct femtosecond-laser writing to fabricate a circularly symmetric waveguide [19,21] to enable the polarization-based filtering of noise inside the integrated structure. The low-damage fabrication process well preserves the coherent properties of the optical transitions and the spin transitions of ^151^Eu^3+^ ions. High-fidelity storage of time-bin qubits is demonstrated by implementing an efficient spin-wave storage protocol based on the noiseless photon echo [26].

## RESULTS

### Experimental setup

**Figure 1.**
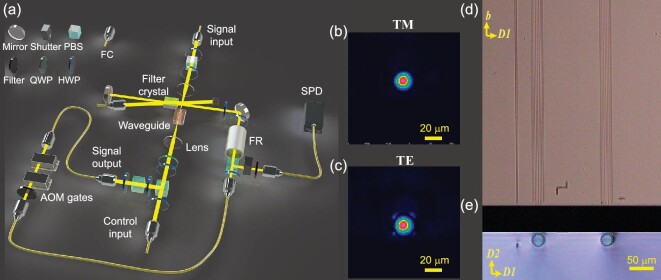
Setups. (a) Schematic of the setup. The signal beam in the TM mode and the control beam in the TE mode are coupled into the laser-written waveguide in a counterpropagating configuration. After storage and retrieval in the waveguide memory, the photons are filtered by temporal gates composed of two cascaded acousto-optic modulators (AOMs), an ultranarrow-band spectral filter based on a double-passed filter crystal and two 1-nm spectral filters, and they are finally detected by a single-photon detector (SPD). FR, Faraday rotator; PBS, polarization beam splitter; HWP, half wavelength plate; QWP, quarter-wave plate; FC, fiber collimator. (b and c) The intensity profiles for guided TM and TE modes with scale bars of 20 *μ*m. The full widths at half maximum of the guided modes are 10.3 × 9.9 *μ*m^2^ (*D*1 × *D*2) for both the TM and TE modes. (d and e) The top and front views of the waveguides with scale bars of 50 *μ*m, respectively. Here *D*1, *D*2 and *b* indicate the crystal axes of the Y_2_SiO_5_ crystal. The waveguide on the right-hand side is used in the experiment.

The substrate is a 0.01% doped ^151^Eu^3+^:Y_2_SiO_5_ crystal with a size of 5 × 4 × 15 mm^3^ along the crystal’s *D*1 × *D*2 × *b* axes. The low doping concentration is chosen here to achieve a small inhomogeneous broadening for spin transitions, which is favorable for spin-wave storage [24–26] and long-lived spin coherence at critical magnetic fields [33,34]. A depressed-cladding waveguide [35–37] is fabricated by a femtosecond-laser-micromachining system; for further details, see Section SI.A within the online supplementary material. The circularly symmetric structure of the depressed-cladding waveguide, also known as the type-III waveguide, enables the transmission of both transverse magnetic (TM) and transverse electric (TE) modes [19]. As shown in panels (b) and (c) of Fig. 1, the guide modes are single modes for both the TM and TE modes and the polarization direction of the TM (TE) mode is along the crystal’s *D*1 (*D*2) axis. Compared with typical experiments performed with a bulk crystal [38], the optical power density can be increased by approximately 300 times due to the spatial confinement of the waveguides. Inspired by the design in angled physical contact fiber connectors, here the transmission direction of the waveguide is deviated from the crystal’s *b* axis by 0.5^○^, to reduce noise caused by direct reflections from the crystal’s surface. The absorption depth for the ^7^F_0_ → ^5^D_0_ transition of Eu^3+^ ions is 1.44 (0.26) for the TM (TE) mode in the waveguide. The properties of the optical transition and spin transitions are well preserved after the fabrication process and detailed characterizations on the waveguide memory can be found in Section SI.C.

The experimental setup is presented in Fig. 1(a). The filtering system consists of polarization beam splitters, AOM gates, 1-nm spectral filters and a filter crystal. The filter crystal is a 0.1% doped ^151^Eu^3+^:Y_2_SiO_5_ crystal with a length of 20 mm and an absorption depth of 6. The waveguide memory and the filter crystal are cooled down to approximately 3.2 K in a closed-cycle cryostat.

### The AFC memory

The atomic frequency comb (AFC) [39] and the noiseless photon echo (NLPE) [26] are two established spin-wave storage protocols developed for solid-state quantum memories. We start our experiment with the implementation of the AFC protocol. The rephasing of the inhomogeneous broadened atomic ensemble is achieved by tailoring the absorption profile into a periodical structure in the AFC memory.

**Figure 2.**
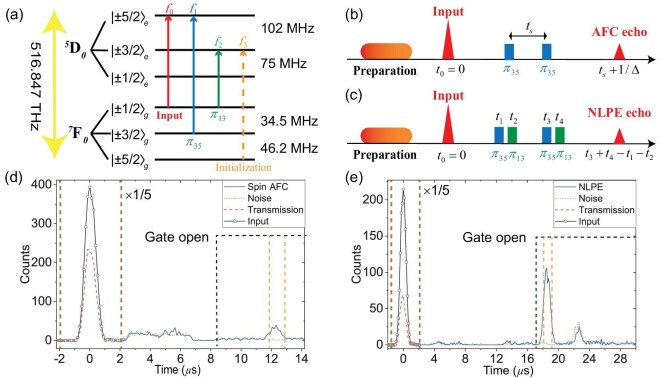
Spin-wave storage of single-photon-level inputs. (a) Level structure of the ^7^F_0_ → ^5^D_0_ transition for ^151^Eu^3+^ ions in the Y_2_SiO_5_ crystal at zero magnetic field. These transitions that are addressed during quantum storage are indicated by *f_x_, x* = 0, 1, 2, 3. (b and c) Simplified time sequence for quantum storage based on the full atomic frequency comb (AFC) and noiseless photon-echo (NLPE) protocols. (d) The photon-counting histograms for the AFC memory with an average number of input photons per pulse μ = 1.40. The input and the transmission are represented by the black solid line with open circles and the red dashed line, respectively. The blue solid line and green dotted line represent the results of the AFC memory with and without inputs, respectively. The black dashed line marks the moment that AOM gates open. The signal-to-noise ratio is 13.1 ± 3.9 with a detection window of 1.05 *μ*s, which is indicated by the orange dashed lines. (e) The photon-counting histograms for NLPE memory with μ = 1.42. The input and the transmission are represented by the black solid line with open circles and the red dashed line, respectively. The blue solid line and green dotted line represent the readout of the NLPE memory with and without inputs, respectively. The signal-to-noise ratio is 60.2 ± 13.8 with a detection window of 1.05 *μ*s. The counts in the input regime, as indicated by the brown dashed lines in (d) and (e), are magnified by 1/5 for visual clarity.

The level diagram and the simplified time sequence are provided in panels (a) and (b) of Fig. 2, respectively. The AFC is prepared in the transition |±1/2〉_*g*_ → |±5/2〉_*e*_, and it has a bandwidth of 2 MHz and a comb interval of 100 kHz, as shown in Fig. S1(c). More details about the preparation process are provided in Section SI.B. The photon counting histogram of the AFC memory is shown in Fig. 2(d). At *t*_0_ = 0, the signal pulse with a duration of 2.0 *μ*s and a full width at half maximum of 0.8 *μ*s is captured by the AFC memory. The input is weak coherent pulses containing μ = 1.40 photons per pulse on average. The two-level AFC storage efficiency is $2.3\%\pm 0.1\%$ at a storage time of 10 *μ*s. To implement spin-wave storage, two optical π_35_ pulses resonant with the transition |±5/2〉_*e*_ → |±3/2〉_*g*_ are employed to reversibly transfer the spin-wave excitations with a controlled spin-wave storage time. In Fig. 2(d), the total integration time is 24.8 h with 125 000 repetitions at a repetition frequency of 1.4 Hz. The spin-wave storage efficiency is $1.6\%\pm 0.1\%$ with a total storage time of 12.2 *μ*s (blue line in Fig. 2(d)). If the detection window is set to 1.05 *μ*s, which is 53% of the total duration and covers 85% of the area of the output mode, the storage efficiency is $1.4\%\pm 0.1\%$ and the signal-to-noise ratio (SNR) is 13.1 ± 3.9.

### The NLPE memory

The storage efficiency of the AFC memory is limited by the spectral tailoring process to prepare the comb, which further reduces the naturally weak absorption of the Eu^3+^:Y_2_SiO_5_ crystal [33,40]. The NLPE protocol is proposed to solve this problem, where higher efficiencies can be obtained by direct optical rephasing so that the original optical absorption can be maximally utilized [21,26,40]. The time sequence for NLPE memory is provided in Fig. 2(c). A flat absorption band with a bandwidth of 2.0 MHz is prepared after the preparation procedure, as shown in Fig. S1(d). The input pulse is similar to that used for the AFC memory with μ = 1.42 photons per pulse. Four optical π pulses are required in the NLPE memory. Two of them, marked as π_35_, are on resonance with the transition |±3/2〉_*g*_ → |±5/2〉_*e*_, and the other two, marked as π_13_, are on resonance with the transition |±1/2〉_*g*_ → |±3/2〉_*e*_. The four π pulses are arranged as π_35_ at time *t*_1_, π_13_ at time *t*_2_, another π_35_ at time *t*_3_ and another π_13_ at time *t*_4_, and the NLPE echo emits at time *t*_3_ + *t*_4_ − *t*_1_ − *t*_2_ [26]. This sequence is different from the original NLPE scheme [26], which is chosen here according to the noise analysis presented in the next section. In Fig. 2(e), the total integration time is 12.9 h with 65 000 repetitions at a repetition frequency of 1.4 Hz. The NLPE storage efficiency is $10.6\%\pm 0.4\%$ at a storage time of 18.6 *μ*s (blue line in Fig. 2(e)). If the detection window is set as 1.05 *μ*s, the storage efficiency is $8.6\%\pm 0.4\%$ and the SNR is 60.2 ± 13.8.

### Noise analysis of AFC and NLPE memories

The noise of a quantum memory can be divided into three parts: coherent noise generated from the memory, incoherent noise generated from the memory and ambient noise caused by the setup other than the memory. Coherent noise is caused by strong control pulses, including free induction decay (FID) and unwanted echoes. Such noise has certain spatial and temporal modes and can be filtered completely in principle. Incoherent noise, i.e. spontaneous emission, is the fundamental noise of an optical quantum memory [41]. If it has a different frequency to the signal then it can be removed by spectral filtering. However, incoherent noise with the same frequency as the signal can be reduced by polarization-based filtering, but cannot be completely removed. Ambient noise has negligible contributions in our experiments through careful optimization of the optical setup. The main sources of both coherent and incoherent noise are the imperfect class cleaning, the residual population at the |±3/2〉_*g*_ state and the imperfect π pulses.

In AFC memory, coherent noise, which includes the FID [42,43] and the two-level photon echo (PE) caused by the π_35_ pulses, can be almost completely removed by the filtering system. Part of the incoherent noise is caused by the residual population at the |±3/2〉_*g*_ state due to imperfect spin initialization. The π_35_ pulses transfer such atomic population to the excited state, leading to indistinguishable spontaneous emission noise. In addition, the truncated rising and falling edges of π_35_ pulses may excite some population at |±1/2〉_*g*_ to the excited state and lead to indistinguishable noise.

In NLPE memory, coherent noise includes the FID and the noisy echoes caused by the π_35_ and π_13_ pulses. Since the π_13_ pulse excites many ions, the two-level PE generated by the pair of π_13_ pulses is strong. To largely separate the two-level echo caused by π_13_ pulses and the signal in the time domain, here the order of the four π pulses is arranged as π_35_, π_13_, π_35_ and π_13_, which is different from that in the original NLPE protocol [26]. Moreover, there is a relatively strong four-level noisy echo located at *t* = 23 *μ*s and other small noisy echoes in Fig. 2(e), which have the same frequency as that of the signal. These noisy echoes can be distinguished from the signal through temporal filtering and a more detailed analysis of these noisy echoes can be found in Section SII. Since the majority of the atomic population is initialized to |±1/2〉_*g*_, most of the incoherent noise would be generated from the excited state |±3/2〉_*e*_, which is different from the excited state |±5/2〉_*e*_ that generates the signal, and the filter crystal can remove this part of the incoherent noise. The atomic population in |±3/2〉_*e*_ may decay into |±3/2〉_*g*_ and those atoms will be excited to |±5/2〉_*e*_ by π_35_ and lead to indistinguishable noise.

All coherent noise can be completely removed in principle, but this represents a long-standing challenge for integrated memories. In quantum memories based on bulk materials [24,26], a small angle between the signal beam and the control beam is introduced to realize spatial filtering. However, such a method is not allowed in integrated memories due to the single spatial mode. Here, our polarization-maintaining waveguide provides polarization filtering to achieve the required suppression of noise. The counter-propagation configuration and the polarization filtering together provide an extinction ratio of ∼83 dB and the filter crystal can provide an extinction ratio of ∼52 dB. The temporal gate provides an extinction ratio of ∼80 dB. In total, the control pulses could be suppressed by ∼215 dB so that they can co-propagate with the single-photon signals inside the same waveguide. The unconditional noise probability (*p*) of the NLPE (AFC) memory is $\sim 0.20\%$ ($\sim 0.15\%$). This represents the probability of detecting a photon within the detection window per experiment without input. In our experiment, coherent noise in the detection windows is negligible due to the powerful filtering system, and the predominant source of noise arises from incoherent factors. The improved SNR of the NLPE memory is mainly due to the enhanced storage efficiency.

### Spin-wave storage of qubits

We further employ the NLPE memory to demonstrate spin-wave storage of the time-bin encoded qubits. The pulse sequence of the preparation and the analysis of the time-bin qubits are shown in Fig. S3. For analysis of superposition states, two phase-controllable (π/2)_13_ pulses are applied to replace the last π_13_ pulse in the NLPE protocol so that the memory itself serves as a temporal beam splitter [24,26].

**Figure 3.**
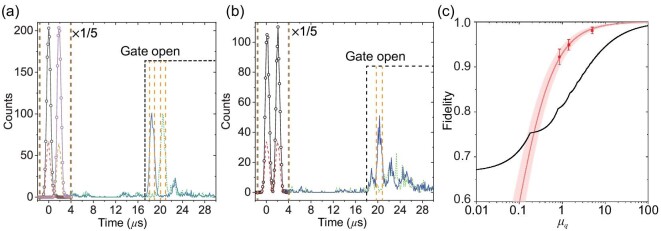
Spin-wave storage of time-bin qubits based on NLPE. (a) The photon-counting histogram for the storage of |*e*〉 and |*l*〉 states with an average number of input photons per qubit μ_*q*_ = 1.42. For the |*e*〉 (|*l*〉) state, the input is represented by the black (pink) solid line with open circles, and the transmission is represented by the red (orange) dashed line, while the NLPE echo is represented by the blue solid (green dotted) line. (b) The photon-counting histogram for the storage of the |*e*〉 + |*l*〉 state with μ_*q*_ = 1.42. To measure the fidelity, two phase-controllable (π/2)_13_ pulses are used to replace the second π_13_ pulse, as shown in Fig. S3(b), to obtain the interference in the center time bin (|*el*〉 + |*le*〉) of the readout [24]. The input and the transmission are represented by the black solid line with open circles and the red dashed line, respectively. The readouts with constructive interference and destructive interference are presented by the blue solid line and the green dotted line, respectively. In (a) and (b) the black dashed line marks the moment that AOM gates open. The detection window is set to 1.05 *μ*s, as indicated by the orange dashed lines. The counts in the input regime, as indicated by the brown dashed lines, are magnified by 1/5 for visual clarity. The photon-counting histogram for the storage of the |*e*〉 + *i*|*l*〉 state is presented in Fig. S3(c). For the |*e*〉, |*l*〉, |*e*〉 + |*l*〉 and |*e*〉 + *i*|*l*〉 states with 60 000 repetitions at a repetition frequency of 1.4 Hz, the total integration times are all 11.9 h. (c) The memory fidelity with variable input levels μ_*q*_. The black line shows the classical bound, which is the maximal fidelity that can be achieved using the classical measure-and-prepare strategy. The red squares show the measured storage fidelity and the red curve is the theoretical prediction of the storage fidelity based on the experimentally measured efficiency and noise. The calculation methods are provided in Sections SIII and SIV.

Four kinds of qubits, |*e*〉, |*l*〉, |*e*〉 + |*l*〉 and |*e*〉 + *i*|*l*〉, are stored and retrieved in the waveguide memory. Panels (a) and (b) of Fig. 3 provide the photon-counting histograms for these experiments with an average number of μ_*q*_ = 1.42 photons per qubit. As shown in Fig. 3(c), the total storage fidelity *F_T_* is $94.9\%\pm 1.2\%$. Here the total fidelity is defined as *F_T_* = (1/3)(*F*_|*e*〉_ + *F*_|*l*〉_)/2 + (2/3)(*F*_|*e*〉 + |*l*〉_ + *F*_|*e*〉 + *i*|*l*〉_)/2, where *F*_|*z*〉_ represents the state fidelity for the corresponding input state of |*z*〉. The fidelities of retrieved qubits with various input levels can be found in Table S1. This result significantly outperforms the maximal achievable fidelity using a classical measure and prepare strategy (83.6%) that considers a storage efficiency of 8.6% and the Poisson statistics of the weak coherent pulse [3,24,26,28]. The storage fidelities *F_T_* are $92.2\%\pm 1.8\%$ and $98.1\%\pm 0.7\%$ for μ_*q*_ = 0.86 and μ_*q*_ = 4.98 photons per qubit, respectively (Fig. 3(c)). All these results are far beyond the classical bound, which demonstrates that this integrated spin-wave optical memory, with the help of the filtering system, operates in the quantum regime.

## CONCLUSIONS

Spin-wave quantum storage has been considered as a principal obstacle towards practical applications of integrated quantum memories [15–21]. Here, based on a comprehensive filtering scheme, we have achieved spin-wave quantum storage using a laser-written waveguide fabricated in a Eu^3+^:Y_2_SiO_5_ crystal. Increasing the optical absorption depth can further improve the storage efficiency. Both AFC and NLPE are echo-based quantum storage protocols that allow temporal multiplexing [26,39] given a large bandwidth and a long optical storage time. A high spatial storage density could also be obtained with three-dimensional waveguide arrays interfaced with optical fiber arrays. Further combining this device with a critical magnetic field to protect the spin coherence [2,33], a long-lived spin-wave quantum memory for light can be constructed. Based on these efforts, we could expect the construction of long-lived transportable quantum memories [2], which could be useful solutions both for long-distance quantum communication and for long-baseline optical interferometry [44]. A weak magnetic field would be sufficient to extend the storage lifetime to tens of milliseconds [28], which can enable extensive applications in multiplexed quantum repeaters [1,13,14,28].

## Supplementary Material

nwae161_Supplemental_File
