# Supplementary material for: Integrated spin-wave quantum memory
Source: Natl Sci Rev. 2024 May 1;11(11):nwae161. doi: 10.1093/nsr/nwae161 (PMC11493096; doi:10.1093/nsr/nwae161)
Supplement: nwae161_Supplemental_File [file nwae161_supplemental_file.pdf]

Supplementary Data for “Integrated Spin-wave Quantum Memory”

I. MATERIALS AND METHODS

A. The device fabrication.

The fabrication of the depressed-cladding waveguide is implemented by a commercial femtosecond-laser-micromachining system (WOPhotonics, Lithuania). This depressed-cladding waveguide, designed as a circular geometry composed of 20 tracks, supports both TM and TE modes [1, 2]. As shown in Fig. 1(e) in the main text, this waveguide is located 17 μm beneath the crystal surface and has a core diameter of 20 μm. During fabrication, an $\times 100$ objective with a numerical aperture of 0.7 focuses the 1030-nm femtosecond laser on the crystal along the $D2$ axis. The pulse duration is 210 fs and the polarization direction of the femtosecond laser is along the crystal's b axis. Inspired by the design in angled physical contact (APC), the processing direction deviates from the crystal's b axis by 0.5 degrees to avoid the noise from the reflection from the crystal surface. The repetition rate of the femtosecond laser is set as 201.9 kHz, and the processing speed is 1 mm/s. Due to the aberration brought by the focal depth from the upper surface of the crystal, we use a graded power scheme to fabricate the waveguide. From deep to shallow below the crystal surface, the energy per pulse is gradually changed from 65 nJ to 61 nJ. Finally, as shown in Fig. 1(b) and Fig. 1(c) in the main text, the guide modes are single modes for both the TM and TE modes, and the polarization direction of the TM (TE) mode is along the crystal's $D1$ ($D2$) axis. The full width at half maximum (FWHM) of the guided modes is 10.3 μm$\times$9.9 μm ($D1 \times D2$) for both TM and TE modes. The birefringence axes direction in the waveguide is not changed compared with the bulk crystal. Compared to a typical experiment performed with a bulk crystal [3], the optical power density of the control modes can be increased by approximately 300 times due to the spatial confinement of waveguides. The integrated operation could also allow easy alignment of the optical setup, which is especially useful in application of long-duration storage where specially-aligned magnetic fields are required. The insertion loss of the waveguide, including coupling loss and transmission loss, is 1.0 (4.0) dB/cm for the TM (TE) mode at 580 nm. For the input with the TM mode, the device efficiency, as defined by transmission between the input and output surface of the crystal waveguide, is 71%. For the control beam with the TM mode, this efficiency is 25%. By removing the crystal waveguide, the measured optical transmission between the front of the cryostat and the fiber collimator of “signal output” (c.f. Fig. 1 in the main text) is 88% and the coupling efficiency of single-mode fiber is 72%. In addition, the transmission efficiency between the “signal output” (c.f. Fig. 1 in the main text) and the final single-photon-detector (SPD) is 27%. The internal storage efficiencies of the AFC and NLPE memories are calculated by the ratio of the readout echo to the input pulse, which has excluded the losses caused by the propagation along the cryostat windows and the waveguides. The system efficiency of the whole setup equals the internal storage efficiency $\times$ the transmission efficiency between the crystal front and the final SPD (12.2%).

Due to the anisotropy of the refractive index changes caused by the femtosecond laser, a perfect balance of insertion losses of the TM and TE modes is difficult to achieve. In our previous work on the site-2 Eu^{3+} ions in Y_2SiO_5 crystals [2], the femtosecond laser irradiates the crystal along the crystal's $D1$ axis and its polarization direction is along the crystal's $D2$ axis. In that case, it is easier to achieve the balance and the optimization of insertion losses of the TM and TE modes. In the current work, we finally chose a waveguide that has a higher efficiency for the TM mode (signal mode) for the experiments. This imbalance between two polarization modes could be optimized by improving the fabrication technique, such as applying a spatial light modulator to correct the aberration of femtosecond pulses and changing the waveguide cross-sectional structure to an ellipse to compensate for the imbalance of the refractive index change [1].

B. Details about the preparation processes for quantum memories.

In the atomic frequency comb (AFC) protocol, the preparation process consists of three parts: the initialization, the spin polarization, and the AFC preparation. Because the hyperfine level spacing is much smaller than the inhomogeneous broadening of the $^7F_0 \rightarrow {}^5D_0$ optical transition (see Fig. 2(a)), nine classes of transitions are addressed by a single-frequency laser pulse with a frequency inside the inhomogeneous broadening. The goal of initialization is to leave only one class of ions within the interested frequency window of the experiment, so that to avoid any disturbance from other classes of ions to the spin-wave memory. One of the methods is that sequentially pump the three spin levels of the ground state for one selected class of ions. Only this selected class of ions can remain in the interested frequency window. Here, we select the three pumping pulses that resonate with the $|\pm 1/2\rangle_g \rightarrow |\pm 5/2\rangle_e$ transition with a frequency of f_0, the $|\pm 3/2\rangle_g \rightarrow |\pm 5/2\rangle_e$ transition with a frequency of f_1, and the $|\pm 5/2\rangle_g \rightarrow |\pm 1/2\rangle_e$ transitions

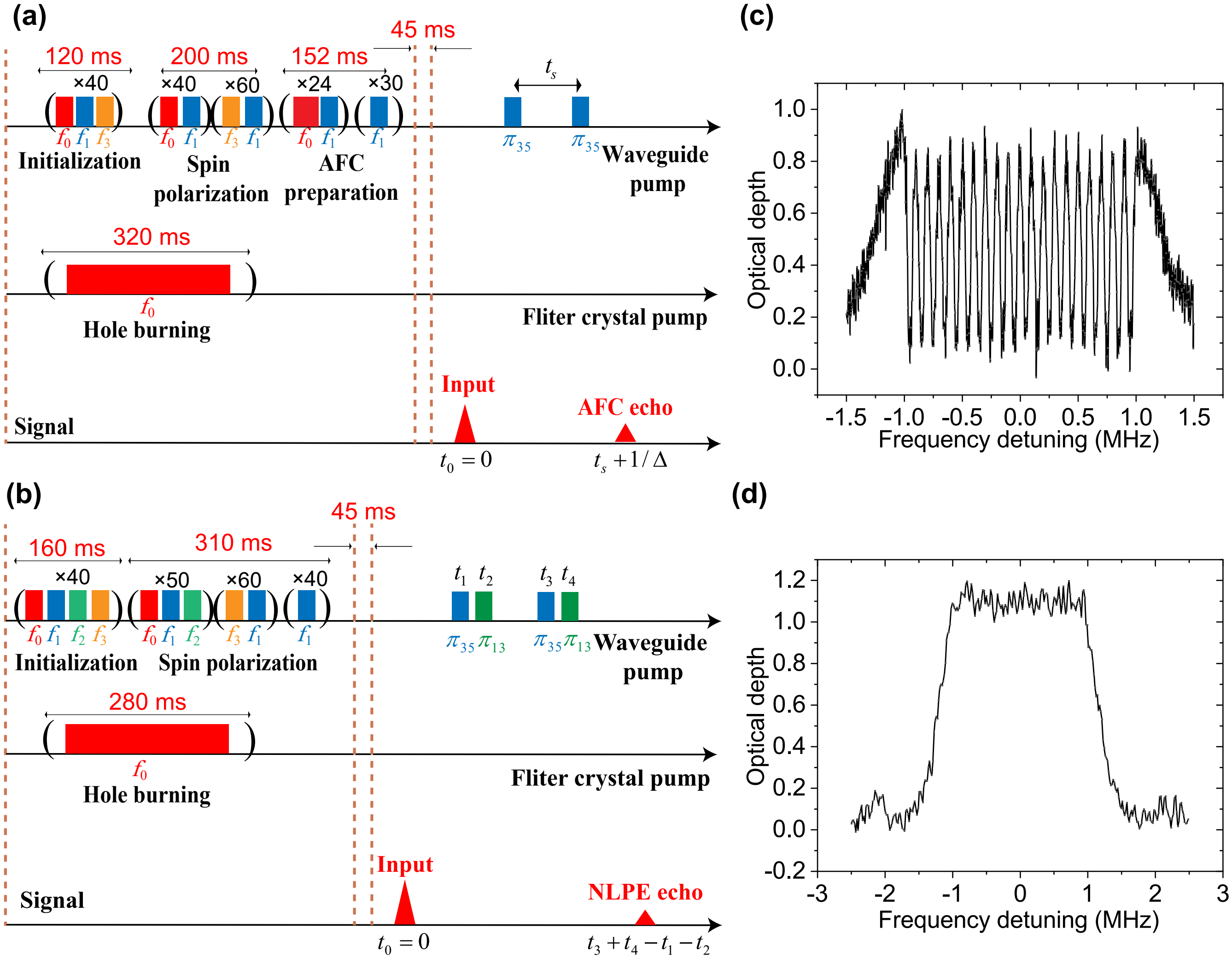

FIG. S1. Pulse sequences of the spin-wave quantum memories. **(a)** and **(b)**, The time sequences of the full atomic frequency comb (AFC) and noiseless photon echo (NLPE) protocols. The labels of the pulses correspond to the different transitions for $^{151}Eu^{3+}$ ions in the Y_2SiO_5 crystal at zero magnetic field. The f_0 represents the $|\pm 1/2\rangle_g \rightarrow |\pm 5/2\rangle_e$ transition, the f_1 represents the $|\pm 3/2\rangle_g \rightarrow |\pm 5/2\rangle_e$ transition, the f_2 represents the $|\pm 5/2\rangle_g \rightarrow |\pm 1/2\rangle_e$ transition, and the f_3 represents the $|\pm 1/2\rangle_g \rightarrow |\pm 3/2\rangle_e$ transition, c.f. Fig. 1 in the main text. **(c)**, The structure of the prepared AFC at the $|\pm 1/2\rangle_g \rightarrow |\pm 5/2\rangle_e$ transition. **(d)**, The structure of the absorption band at $|\pm 1/2\rangle_g \leftrightarrow |\pm 5/2\rangle_e$ transition for the NLPE memory.

with a frequency of f_3. The initialization contains the three chirp pulses with a duration of 1 ms, a bandwidth of 4 MHz, and center frequencies of f_0, f_1, and f_3. These pulses are repeated 40 times with a total time of 120 ms. A single class of ions is selected with a bandwidth of 4 MHz after the initialization. The time sequence of the spin polarization consists of two parts. The first part is polarizing all ions into the $|\pm 5/2\rangle_g$ state by applying chirp pulses with center frequencies of f_0 and f_1. The second part is polarizing all ions into the $|\pm 1/2\rangle_g$ state and clearing the $|\pm 3/2\rangle_g$ state by applying chirp pulses with center frequencies of f_3 and f_1. These pulse sequences of spin polarization could efficiently burn away the unwanted classes of ions in the interested spectral window. We employ the parallel method [3] to create the periodic absorption structure of the AFC. Chirp pulses with a center frequency of f_1 are employed to keep the $|\pm 3/2\rangle_g$ state empty of population, which is essential to reduce the noise in the spin-wave quantum storage. Meanwhile, the spectral preparation process for the filter crystal maintains a 1.8-MHz transparent pit with a center frequency of f_0. The structure of the AFC is detected by weak chirp pulses with a duration of 30 ms, a center frequency of f_0 and a bandwidth of 3 MHz (see Fig. S1(c)). In AFC memory, two optical π pulses resonant with the transition $|\pm 5/2\rangle_e \rightarrow |\pm 3/2\rangle_g$, marked as π_{35} are employed to reversibly transfer the spin-wave excitations with a controlled spin-wave storage time. The π_{35} pulse is an adiabatic pulse [4] with a bandwidth of 2.8 MHz, a peak

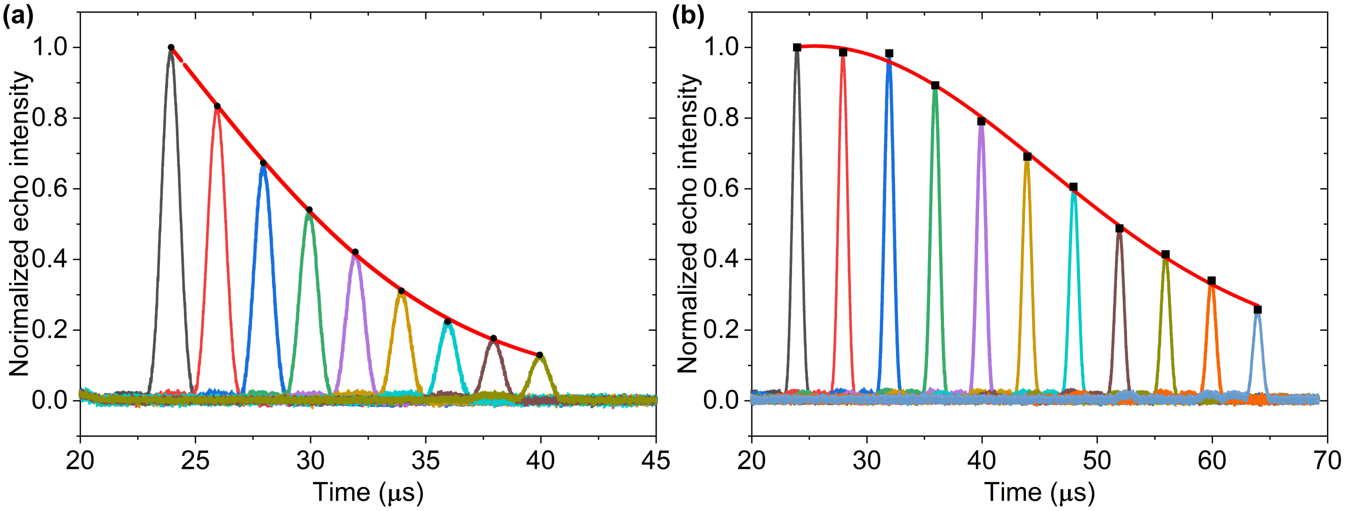

FIG. S2. Characterization of the inhomogeneous broadening of spin transitions of $^{151}Eu^{3+}$ inside the waveguide and long-lived spin-wave storage with NLPE. The black dots represent the area of the NLPE echo inside a 2-μs integral window, and the red lines are the Gaussian fit to the data according to Eq. S1. The original data of the readout traces are also presented for reference. **(a)**, Dependence of the echo intensity on the delay $t_{42} = t_4 - t_2$ by changing $t_4 - t_3$ while keeping $t_2 - t_1$ and $t_3 - t_2$ unchanged. **(b)**, Dependence of the echo intensity on the delay $t_{31} = t_3 - t_1$ by changing $t_2 - t_1$ while keeping $t_3 - t_2$ and $t_4 - t_3$ unchanged.

power of 44 mW, and a duration of 2.2 μs. Compared with typical experiments performed with bulk crystals [3], the required peak power is reduced by 13.6 times and the π-pulse length is reduced by 7 times.

In the noiseless photon echo (NLPE) protocol, the preparation process consists of two parts: initialization and spin polarization (see Fig. S1(b)). Different from that for AFC protocol where only three spectral windows are initialized, here we need to initialize four frequency windows to include the new one which has a center frequency of f_2, which is resonant with the $|\pm 1/2\rangle_g \rightarrow |\pm 3/2\rangle_e$ transition. The spin polarization consists of two steps. The first step is polarizing all ions into the $|\pm 5/2\rangle_g$ state by applying chirp pulses with center frequencies of f_0, f_1, and f_2. The second step is polarizing all ions into the $|\pm 1/2\rangle_g$ state and keeping the $|\pm 3/2\rangle_g$ state empty by applying chirp pulses with center frequencies of f_2 and f_1. The clearing of the $|\pm 3/2\rangle_g$ state is crucial for the suppression of the spontaneous emission noise in NLPE memory. The spectral preparation process for the filter crystal is the same as that for the AFC experiments. The absorption profile after the preparation process is shown in Fig. S1(d). In NLPE memory, four optical π pulses are required in the NLPE memory. Two of them, marked as π_{35}, are in resonance with the transition $|\pm 3/2\rangle_g \rightarrow |\pm 5/2\rangle_e$, and the other two of them, marked as π_{13}, are in resonance with the transition $|\pm 1/2\rangle_g \rightarrow |\pm 3/2\rangle_e$. The π_{35} (π_{13}) pulses are adiabatic pulses with a bandwidth of 2.9 (2.8) MHz, a peak power of 44 (49) mW, and a duration of 2.2 (2.1) μs.

C. Characterization of the $^{151}Eu^{3+}$ ions inside this depressed-cladding waveguide.

The coherent properties of Eu^{3+} ions inside the waveguide are crucial for their applications as quantum memories. Here, we measure the optical inhomogeneous broadening of the $^7F_0 \rightarrow {}^5D_0$ transition, the optical coherence time, and the inhomogeneous broadening of spin transitions to characterize the $^{151}Eu^{3+}$ ions inside this depressed-cladding waveguide.

Optical inhomogeneous broadening is determined by absorption measurements. In the waveguide (bulk) region, the FWHM of the $^{151}Eu^{3+}$ transition is 1.1 ± 0.1 (0.7 ± 0.1) GHz, and the peak absorption depth is 1.44 (1.87). A slight broadening is induced by the fabrication of the waveguide. The optical coherence time (T_2) is measured by the two-pulse photon echo [5]. In the waveguide (bulk) region, we measure $T_2 = 258 \pm 14$ (284 ± 29) μs when the peak power of the incident pulse is 116 (602) μW before the cryostat. The fabrication process has little effect on the optical coherence lifetime.

To characterize the spin coherent properties of the waveguide, we measure the inhomogeneous broadening of spin transitions by implementing the NLPE memory. The four π pulses are arranged as: π_{35} at time t_1, π_{13} at time t_2, π_{35} at time t_3, and π_{13} at time t_4, which is slightly different from the original NLPE scheme [6]. At $t_0 = 0$, we incident

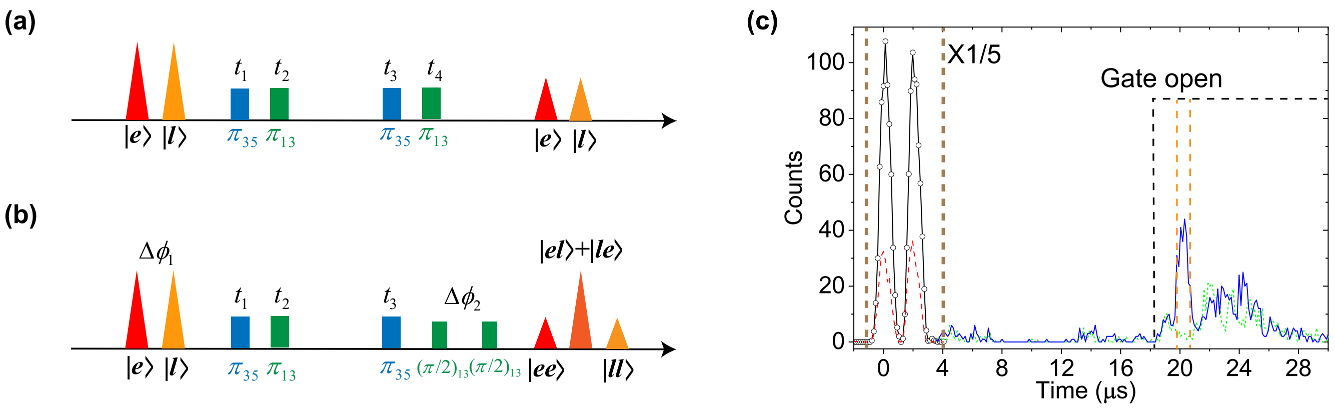

FIG. S3. The NLPE storage of the time-bin qubits. **(a)**, The time sequence for preparing and measuring the eigen time-bin qubits. **(b)**, The time sequence for preparing and measuring the superposition time-bin qubits. **(c)**, The photon-counting histogram for the storage of the $|e\rangle + i|l\rangle$. state with an average number of input photons per qubit $\mu_q = 1.42$. The input and the transmission are represented by the black solid line with hollow circles and the red dashed line, respectively. The readouts with constructive interference and destructive interference are presented by the blue solid line and the green dotted line, respectively. The black dashed line marks the moment that the AOM gates open. The detection window is set to 1.05 μs as indicated by the orange dashed lines. The counts in the input regime, as indicated by the brown dashed lines, are magnified by 1/5 for visual clarity. The total integration time is 11.9 hours with 60000 repetitions at a repetition frequency of 1.4 Hz.

the signal pulse with the frequency of f_0. At time t_1, the first π pulse (π_{35}) transfers the coherence between the $|\pm 3/2\rangle_g$ and $|\pm 5/2\rangle_e$ states into the spin coherence of $|\pm 1/2\rangle_g \leftrightarrow |\pm 3/2\rangle_g$ in ground state. At time t_2, the second π pulse (π_{13}) transfers the spin coherence between the $|\pm 1/2\rangle_g$ and $|\pm 3/2\rangle_g$ states into the optical coherence of $|\pm 3/2\rangle_g \leftrightarrow |\pm 3/2\rangle_e$. However, due to the signal beam and control beam in a counterpropagating configuration, the mismatch of wavevectors silences the four-level photon echo [7] at $t_2 + t_1$. Next, at time t_3, the third π pulse (π_{35}) transfers the optical coherence into the spin coherence of $|\pm 3/2\rangle_e \leftrightarrow |\pm 5/2\rangle_e$ in excited state. At time t_4, the fourth π pulse (π_{13}) transfers the spin coherence into the optical coherence of $|\pm 1/2\rangle_g \leftrightarrow |\pm 5/2\rangle_e$. Finally, the condition of the spatial phase-matching is fulfilled and the NLPE echo is retrieved in the same direction as the input signal [6]. According to the sequence of π pulses in our experiment, the efficiency of NLPE memory can be recalculated as [6]

$$\eta = d^2 e^{-d} \left(\eta_{\text{control}}\right)^4 e^{-\gamma_{13}{}^2 t_{31}{}^2/\left(2\ln(2)/\pi^2\right)} e^{-\gamma_{\bar{3}\bar{5}}{}^2 t_{42}{}^2/\left(2\ln(2)/\pi^2\right)} e^{-2\gamma\cdot t_{42}}, \tag{S1}$$

where d is the absorption depth, $\eta_{control}$ is the average transfer efficiency of the four π pulses, γ_{13} is the inhomogeneous broadening of the spin transition $|\pm 1/2\rangle_g \to |\pm 3/2\rangle_g$, $\gamma_{\bar{3}\bar{5}}$ is the inhomogeneous broadening of the spin transition $|3/2\rangle_\mathrm{e} \to |5/2\rangle_\mathrm{e}$, γ is the effective optical decoherence rate, and t_{xy} is the time interval between the y_{th} and the x_{th} π pulses. Fig. S2 presents that the NLPE efficiencies with varying t_{31} and t_{42}. The efficiencies are measured with classical light as the input and represented with the echo area. According to data fit based on Eq. S1, these parameters of the waveguide memory are listed here: $\gamma_{\bar{3}\bar{5}} = 23 \pm 1$ kHz, $\gamma_{13} = 7.3 \pm 1$ kHz, $\gamma = 8 \pm 4$ kHz, and the average efficiency of the control pulse is 83% as estimated from the fit. Compared with the results obtained in the bulk material [6], these parameters are approximately unchanged, which indicates that the fabrication process introduces negligible damages to the material. This property is of crucial importance for further applications in transportable quantum memories where the spin inhomogeneous broadening could put a strong limit on the achievable coherence lifetime [8, 9].

D. Details about the spin-wave storage of qubits.

The time sequence for preparing and measuring the time-bin qubits is shown in Fig. S3. Manipulating the relative weight and the relative phase ($\Delta\phi_1$) of $|e\rangle$ and $|l\rangle$ states could prepare arbitrary time-bin qubits. The qubits in eigen states are stored with the standard NLPE memory (see Fig. S3(a)) and the qubits in superposition states are measured by the two phase-controllable $(\pi/2)_{13}$ pulses (see Fig. S3(b)). The interval between two $(\pi/2)_{13}$ pulses is set as the same as that of the two input pulses, so that an interference can be obtained in the middle of the readout [6]. The $(\pi/2)_{13}$ pulses are adiabatic with a bandwidth of 1.8 MHz, a peak power of 44 mW and a duration of 1.5

TABLE S1. Fidelities of retrieved qubits with various input levels.

Average photons per qubit μ_q	$F_{\|e\rangle}$	$F_{\|l\rangle}$	$F_{\|e\rangle+\|l\rangle}$	$F_{\|e\rangle+i\|l\rangle}$	F_T
0.86	$95.1 \pm 1.1\%$	$95.6 \pm 1.0\%$	$89.7 \pm 2.2\%$	$91.6 \pm 2.0\%$	$92.2 \pm 1.8\%$
1.42	$98.4 \pm 0.5\%$	$98.3 \pm 0.5\%$	$93.2 \pm 1.5\%$	$93.1 \pm 0.5\%$	$94.9 \pm 1.2\%$
4.98	$99.2 \pm 0.3\%$	$99.1 \pm 0.4\%$	$98.0 \pm 0.9\%$	$97.2 \pm 1.0\%$	$98.1 \pm 0.7\%$

μs. The fidelity of four kinds of qubits $|e\rangle$, $|l\rangle$, $|e\rangle + |l\rangle$, and $|e\rangle + i|l\rangle$ is employed to characterize the fidelity of the time-bin-qubit memories. The average photons per qubit μ_q is defined as the average number of photons per qubit before the cryostat. The total fidelity is defined as $F_T = \frac{1}{3}\frac{F_e+F_l}{2} + \frac{2}{3}\frac{F_{e+l}+F_{e+il}}{2}$, where F_z represents the state fidelity for the input state $|z\rangle$. The memory fidelities for various input levels are shown in Tab. S1.

Compared to the recent demonstration of AFC [10] and NLPE [6] memories in bulk crystals, this integrated quantum memory has a comparable performance in terms of both efficiency and fidelity. There are several methods to improve the storage performance of the current device. The storage efficiency is primarily limited by the low absorption. A higher efficiency could be obtained by using a longer crystal sample or an impedance-matched cavity [11, 12]. The filtering system could be upgraded to reduce optical losses and to provide a better extinction ratio with longer $^{153}Eu^{3+}$:Y_2SiO_5 filter crystals so that a higher fidelity could be expected.

E. Additional details of the experiment setup.

Single photon detector. The single-photon detection module (Excelitas, SPCM-AQRH-46-FC) has a detection efficiency of 65% at 580 nm, and the dark count rate is approximately 12 Hz.

Single photon counter. The time-correlated single photon counting system is HydraHarp 400 from PicoQuant GmbH.

Narrow linewidth laser. The frequency-doubled semiconductor laser at 516.847 THz (TA-SHG, Toptica) is locked by a Fabry-Perot cavity (ATFilms) with a fineness of 40000. After stabilization using the Pound-Drever-Hall technique [13], the final linewidth of the laser is approximately 0.4 kHz.

Cryostat. The cryostat is a closed-cycle cryostat from Montana Instruments, with a sample temperature of approximately 3.2 K. Three-axis cryogenic nanopositioners (Attocube) are employed for the coupling of light into the waveguide. To implement long-lived spin-wave storage at critical magnetic fields in the future, one will need a closed-cycle cryostat equipped with a superconducting magnet [14]. The current working temperature is sufficient, since both the optical coherence lifetime and spin coherence lifetime are independent with temperature at the temperature range of 1.5 K to 5 K [15, 16]. Given the intense research efforts on compact cryogenic systems, especially those for space missions, we expect a portable quantum memory could be feasibly implemented in the near future.

II. ADDITIONAL DETAILS ABOUT THE COHERENT NOISE IN NLPE MEMORY.

This section provides a detailed analysis of the noisy echoes presented in Fig. 2(e) of the NLPE memory. There are two kinds of coherent noisy echoes, namely 2-level and 4-level photon echoes (PE). The readout time of any 2-level PE follows the formula $t = 2t' - t_i$, where t' represents the incident time of the first π pulse and t_i represents the incident time of the input pulse. In contrast, the readout time of any 4-level PE follows $t = (t' + t'') - t_i$ [17], where $t'(t'')$ represents the incident time of the first (second) π pulse. Thus, by modifying the time sequences of the π pulses, it is possible to distinguish between the 2-level and 4-level PEs. These echoes are coherent noise and can be intentionally enhanced by adjusting the polarization maintenance of the optical path. An analysis of these echoes is then performed in both the temporal and spectral domains. Figures S4(a) and S4(b) show the changes in the readout time of noisy echoes by altering the time sequences of π pulses. Figure S5(a) shows the results of a sketch of the noisy echoes for the new NLPE sequence, where the noisy echoes consist of three 4-level photon echoes (PE) and one 2-level PE.

At the beginning of the test, the atomic population is polarized to the $|\pm 1/2\rangle_g$ level, as in the NLPE memory. Based on the pulse sequence of '$\pi_{35} \cdot 0$-π_{13}-5 μs-$\pi_{35} \cdot 0$-π_{13}' (the orange line in Fig. S4(b)), it is evident that a 2-level PE is resulting from the pair of π_{13} pulses. Note that here $\pi \cdot 0$ means that this pulse amplitude is set to zero. In contrast, for the pulse sequence of 'π_{35}-$\pi_{13} \cdot 0$-5 μs-π_{35}-$\pi_{13} \cdot 0$' (the blue-green line in Fig. S4(b)), the 2-level PE from the pair of π_{35} pulses is small enough to be ignored. This pulse sequence also allows us to deduce that the coherent noise in AFC memory is negligible. As shown in Fig. S5, the new NLPE sequence switches the order of the second π_{35} pulse and the second π_{13}pulse in the original sequence [6], which delays the 2-level PE caused by π_{13} pulses and

provides longer noise-free windows for more temporal modes. In principle, this 2-level PE can be eliminated entirely by implementing better frequency and polarization-based filtering, without any negative impact on quantum storage or temporal multiplexing.

Based on the pulse sequences shown in Fig. S4, temporal analysis reveals that the highest noisy echo at t=22.6 μs includes two 4-level PE. Besides, the pulse sequence indicated by '$\pi_{35}\cdot$ 0-π_{13}-5 μs-π_{35}-π_{13}' (the red line in Fig. S4(b)) also has a noisy echo at t=∼22.6 μs. This indicates that two sequences, whether or not the first π_{35} pulses are present, can generate noisy echoes at similar times. So, the noisy echo at 22.6 μs may consist of two separate noisy echoes, namely the 4-level PE-1 and the 4-level PE-2 in Fig. S5(a). What's more, in the time sequence of 'π_{35}-π_{13}-5 μs$-\pi_{35}$-2 μs-π_{13}' (the blue line in Fig. S4(a)), a 4-level noisy echo is present at t=∼26.5 μs, marked as 4-level PE-3. The 4-level PE-3 is insignificant due to overlap with the 2-level PE at t=∼24.5 μs in the pulse sequence of 'π_{35}-π_{13}-5 μs$-\pi_{35}$-π_{13}' (the black line in Fig. S4(a)). By employing the filter crystal as a tunable spectral analyzer, we find that these 4-level noisy echoes have the same center frequency (f_0) as that of the signal, and the bandwidth is identical to the transparent window. Due to the short π pulses with a duration of 2.1 μs for π_{13} and 2.2 μs for π_{35}, there are truncated rising and falling edges which could lead to wideband excitation of the atoms. We recently found that optimizing the rising edge of the π pulse reduces the 4-level noisy echoes. We infer that the rising edge of the first π_{35} pulse excites the ions at f_0, which is read out as the 4-level PE-1 at f_0 at t=∼22.6 μs. The 4-level PE-3 is generated by the rising edge of the first π_{13} pulse, which can excite the remaining population in the $|\pm 3/2\rangle_g$ state with the transition of $|\pm 3/2\rangle_g \to |\pm 3/2\rangle_e$. The optical coherence is transferred to the $|\pm 1/2\rangle_g \leftrightarrow |\pm 5/2\rangle_e$, and the 4-level PE-3 is read out at t=∼24.6 μs. Similarly, the 4-level PE-2 is generated by the falling edge of the first π_{13} pulse and emits at t=∼22.6 μs. In Ref. [18], a similar noisy echo called the off-resonant echo was found in the AFC memory. These noisy echoes can be distinguished from the signal by temporal gating and better polarization filtering, as they are coherent noise with well-defined optical modes.

The filter crystal is a 0.1% doped $^{151}Eu^{3+}$:Y_2SiO_5 crystal with a length of 20 mm and an absorption depth of 6. The double-pass filter crystal can provide an extinction ratio of $\sim$ 52 dB for noise with frequencies different from the signal. However, because the filter crystal and the waveguide have the same energy level structure, the rejection ratio of π_{13} will be reduced by 1/3. Because when we burn a spectral hole at the frequency f_0 of the signal mode for the filter crystal, a spectral hole will be generated at the frequency of π_{13} pulses. If we assume that the population of all classes of ions is the same, the optical depth of this spectral hole accounts for 1/3 of the total optical depth. This problem can be solved by replacing the filter crystal with the isotope of $^{153}Eu^{3+}$ [19]. The total extinction ratio of the control pulses is expected to be $\sim$ 215 dB. However, there are still some detectable residual counts of the control pulses in Fig. 2(d) and Fig. 2(e) in the main text. They mainly come from the scattered light from the waveguide that leaks directly into the optical path of the filter crystal.

In the noise test shown in Fig. S4, the polarization extinction ratio was deliberately decreased, which greatly increased the proportion of coherent noise relative to incoherent noise. Therefore the amount of noisy echo data accumulated in Fig. S4 is larger than that in Fig. 2(e) in the main text.

III. THE THEORETICAL PREDICTION OF THE STORAGE FIDELITY.

Given the unconditional noise p, which is the probability of detecting a photon in the detection window per experiment without input, the storage fidelity F for single-photon-level inputs can be calculated by

$$F = S_{max}/(S_{max} + S_{min}), \tag{S2}$$

where $S_{max}(S_{min})$ is the photon counts measured with the constructive interference (destructive interference). Considering the storage efficiency η_M and the experiment times N, we can get

$$S_{\max} = (\mu_q \eta_M \cdot F_c + p)\, N, S_{\min} = [\mu_q \eta_M \cdot (1 - F_c) + p]\, N, \tag{S3}$$

where μ_q is the average number of photons per qubit, F_c is the classical fidelity without considering the unconditional noise p. Then substitute Eq. S3 into Eq. S2, the storage fidelity $F(\mu_q)$ at single photon level can be expressed as

$$F(\mu_q) = \frac{\mu_q \eta_M \cdot F_c + p}{\mu_q \eta_M + 2p} = \frac{F_c + p/(\mu_q \eta_M)}{1 + 2p/(\mu_q \eta_M)}, \tag{S4}$$

Here we assume $F_c = 1$ according to experiments performed with classical input. According to Eq. S4, the storage fidelity $F_z(\mu_q)$, where $z = |e\rangle$, $|l\rangle$,$|e\rangle + |l\rangle$, and $|e\rangle + i|l\rangle$ states, can be obtained by the experimentally determined

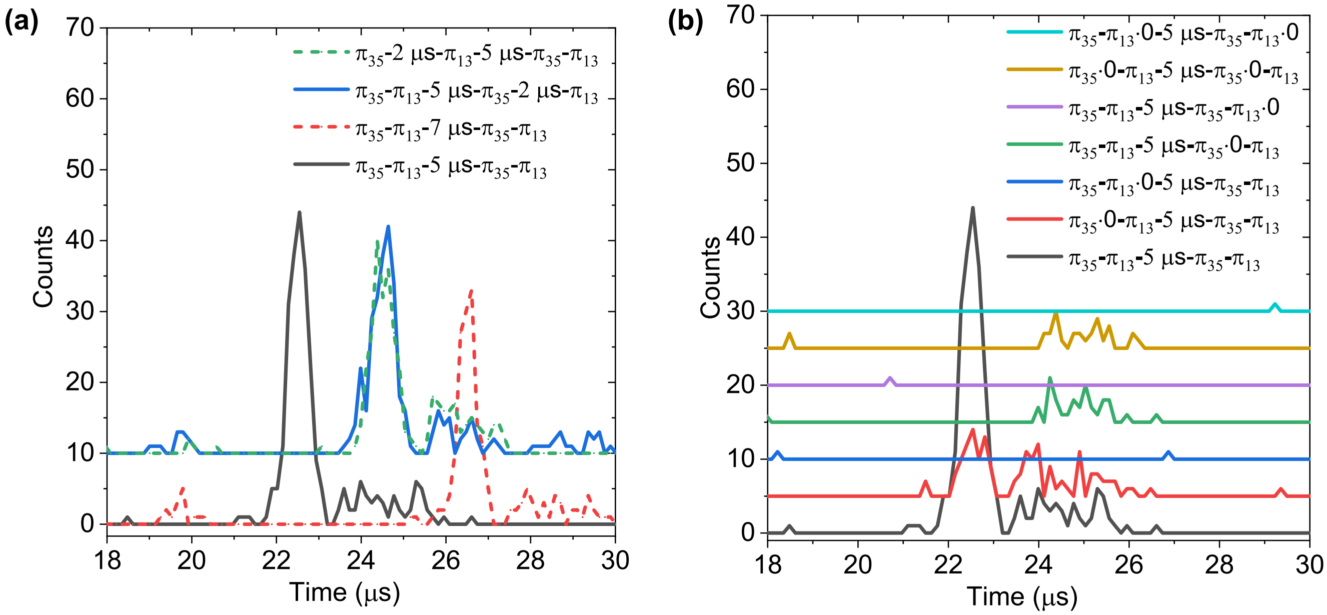

FIG. S4. The analysis of the coherent noise in NLPE memory. The coherent noise has been deliberately increased by adjusting the polarization maintenance of the optical setup. Since the integration time for each pulse sequence is 214 seconds with 300 repetitions at a repetition frequency of 1.4 Hz, the incoherent noise can be ignored. **(a)**, The change of readout time of the noisy echoes with the different time sequences of the π pulses. Take the black line for example, the sequence means that the first π pulse is π_{35}, the second π pulses (π_{13}) is incident without delay after the first π pulse, the third π pulse (π_{35}) is incident at 5 μs after the end of the second pulse, and the last π pulse (π_{13}) is incident without delay after the third π pulse. The blue solid line and green dashed line are offset by 10 counts for visual clarity. In **(a)** and **(b)**, the data shown with a black line corresponds to the noisy echoes in Fig. 2(e) in the main text. **(b)**, The noisy echoes disappear if one or two of the π pulses are set to the amplitude of 0 in the pulse sequences. For visual clarity, lines of different colors are offset by 5 counts, following the legend's order.

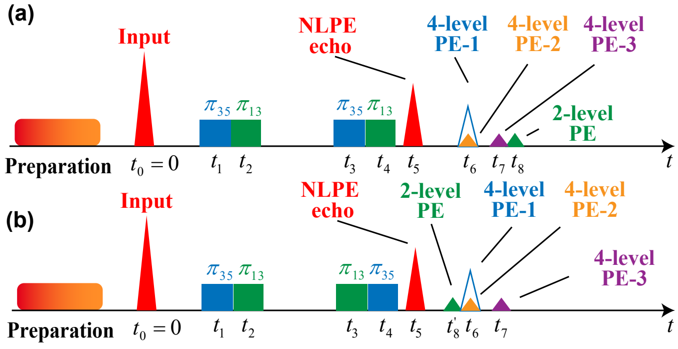

FIG. S5. A sketch of the noisy echoes for **(a)** the new NLPE sequence and **(b)** the original NLPE sequence [6].

efficiency η_{Mz} and the noise p_z. The total storage fidelity $F_T(\mu_q)$, as calculated from $F_z(\mu_q)$, is shown with red curve and red shadowed area in Fig. 3(c) in the main text. The efficiency η_{Mz} and noise p_z are determined with experiments at $\mu_q = 1.42$. Based on the errors in measuring η_{Mz} and p_z, the one standard deviation error of $F_T(\mu_q)$ is shown with red shadowed area in Fig. 3(c) in the main text.

IV. THE CLASSICAL BOUND OF STORAGE FIDELITY.

To verify the quantum behavior of the memory, we simulate the maximal fidelity that can be obtained by using the classical measure-and-prepare strategy, in which a classical device measures the qubit and prepares a new qubit based on the result of the measurement. For a classical memory, the maximum fidelity obtainable is [20]

$$F_c = (n+1)/(n+2), \tag{S5}$$

where n is the number of input photons per qubit. Considering the weak coherent pulse used in the experiment, the number of photons per qubit n satisfies the Poisson distribution,

$$P(\mu_q, n) = e^{(-\mu_q)}\mu_q^n/n!. \tag{S6}$$

Under this condition, the maximum fidelity of the classical memory is [21]

$$F_{\mathrm{CP}}(\mu_q) = \sum_{n\geq 1}^{+\infty} F_c \cdot P(\mu_q, n) / (1 - P(\mu_q, 0)), \tag{S7}$$

where $n \geq 1$ and n are integers. Considering the efficiency $\eta_M < 1$ of the actual memory, we can obtain higher classical fidelity by only considering those inputs that have a large number of photons per qubit n [22, 23]. The maximum fidelity for $\eta_M < 1$ can be written as [21, 23, 24]

$$F_C(\mu_q, \eta_M) = \left[\left(\frac{n_{\min}+1}{n_{\min}+2}\right)\gamma + \sum_{n\geq n_{\min}+1}\frac{n+1}{n+2}P(\mu_q, n)\right] / \left(\gamma + \sum_{n\geq n_{\min}+1} P(\mu_q, n)\right), \tag{S8}$$

where n_{min} is the minimum of i that satisfies $\sum_{n\geq i+1} P(\mu_q, n) \leq (1 - P(\mu_q, 0))\eta_M$, which η_M is the memory efficiency, and parameter γ satisfies $0 \leq \gamma \leq P(\mu_q, n_{min})$ and $\gamma = (1 - P(\mu_q, 0))\eta_M - \sum_{n\geq n_{min}+1} P(\mu_q, n)$. According to Eq. S8, the maximum fidelity $F_{\mathrm{C}}(\mu_q, \eta_M)$ is shown with black line in Fig. 3(c) in the main text. This result has taken into account the internal storage efficiency η_M of 8.6% and the statistics of the input coherent states.

[1] Skryabin N, Kalinkin A, Dyakonov I *et al.* Femtosecond laser written depressed-cladding waveguide 2×2, 1×2 and 3×3 directional couplers in Tm^{3+}:YAG crystal. *Micromachines* 2020; **11**: 1.

[2] Zhu TX, Liu C, Jin M *et al.* On-demand integrated quantum memory for polarization qubits. *Phys Rev Lett* 2022; **128**: 180501.

[3] Jobez P, Timoney N, Laplane C *et al.* Towards highly multimode optical quantum memory for quantum repeaters. *Phys Rev A* 2016; **93**: 032327.

[4] Gündoğan M, Ledingham PM, Kutluer K *et al.* Solid state spin-wave quantum memory for time-bin qubits. *Phys Rev Lett* 2015; **114**: 230501.

[5] Tittel W, Afzelius M, Chaneliere T *et al.* Photon-echo quantum memory in solid state systems. *Laser Photonics Rev* 2010; **4**: 244–267.

[6] Ma YZ, Jin M, Chen DL *et al.* Elimination of noise in optically rephased photon echoes. *Nat commun* 2021; **12**: 4378.

[7] Beavan SE, Ledingham PM, Longdell JJ *et al.* Photon echo without a free induction decay in a double-λ system. *Optics Letters* 2011; **36**: 1272–1274.

[8] Zhong M, Hedges MP, Ahlefeldt RL *et al.* Optically addressable nuclear spins in a solid with a six-hour coherence time. *Nature* 2015; **517**: 177–180.

[9] Ma YZ, Lv YC, Yang TS *et al.* Monte carlo simulation of the nuclear spin decoherence process in Eu^{3+}:Y_2SiO_5 crystals. *Phys Rev B* 2023; **107**: 014310.

[10] Ortu A, Holzäpfel A, Etesse J *et al.* Storage of photonic time-bin qubits for up to 20 ms in a rare-earth doped crystal. *npj Quantum Inf* 2022; **8**: 29.

[11] Sabooni M, Li Q, Kröll S *et al.* Efficient quantum memory using a weakly absorbing sample. *Phys Rev Lett* 2013; **110**: 133604.

[12] Jobez P, Usmani I, Timoney N *et al.* Cavity-enhanced storage in an optical spin-wave memory. *New Journal of Physics* 2014; **16**: 083005.

[13] Black ED. An introduction to pound-drever-hall laser frequency stabilization. *American Journal of Physics* 2001; **69**: 79–87.

[14] Ma Y, Ma YZ, Zhou ZQ *et al.* One-hour coherent optical storage in an atomic frequency comb memory. *Nat commun* 2021; **12**: 2381.

[15] Arcangeli A, Macfarlane RM, Ferrier A *et al.* Temperature dependence of nuclear spin coherence in Eu^{3+} : y_2Sio_5. *Phys Rev B* 2015; **92**: 224401.

[16] Könz F, Sun Y, Thiel C *et al.* Temperature and concentration dependence of optical dephasing, spectral-hole lifetime, and anisotropic absorption in eu 3+: Y 2 sio 5. *Phys Rev B* 2003; **68**: 085109.
[17] Beavan SE, Ledingham PM, Longdell JJ *et al.* Photon echo without a free induction decay in a double-λ system. *Optics Letters* 2011; **36**: 1272–1274.
[18] Timoney N, Usmani I, Jobez P *et al.* Single-photon-level optical storage in a solid-state spin-wave memory. *Phys Rev A* 2013; **88**: 022324. PRA.
[19] Jin M, Ma YZ, Zhou ZQ *et al.* A faithful solid-state spin-wave quantum memory for polarization qubits. *Sci Bull* 2022; **67**: 676–678.
[20] Massar S and Popescu S. Optimal extraction of information from finite quantum ensembles. *Phys Rev Lett* 1995; **74**: 1259–1263.
[21] Laplane C, Jobez P, Etesse J *et al.* Multiplexed on-demand storage of polarization qubits in a crystal. *New Journal of Physics* 2015; **18**: 013006.
[22] Specht HP, Nölleke C, Reiserer A *et al.* A single-atom quantum memory. *Nature* 2011; **473**: 190–193.
[23] Gündoğan M, Ledingham PM, Almasi A *et al.* Quantum storage of a photonic polarization qubit in a solid. *Phys Rev Lett* 2012; **108**: 190504.
[24] Yang TS, Zhou ZQ, Hua YL *et al.* Multiplexed storage and real-time manipulation based on a multiple degree-of-freedom quantum memory. *Nat commun* 2018; **9**: 3407.
